# Vitamin E protects against extraskeletal calcification in uremic rats fed high fat diets

**DOI:** 10.1186/s12882-017-0790-4

**Published:** 2017-12-28

**Authors:** Rafael Rios, Ana I. Raya, Carmen Pineda, Mariano Rodriguez, Ignacio Lopez, Escolastico Aguilera-Tejero

**Affiliations:** 1 Departamento Medicina y Cirugia Animal, Universidad de Cordoba, Campus Universitario Rabanales, Ctra Madrid-Cadiz km 396, 14014 Cordoba, Spain; 2 Instituto Maimonides de Investigacion Biomedica de Cordoba (IMIBIC), Hospital Universitario Reina Sofia, Universidad de Cordoba, Avda Menéndez Pidal s/n, 14004 Cordoba, Spain

**Keywords:** Vascular calcification, Uremia, High-fat diet, Vitamin E

## Abstract

**Background:**

High fat diets are implicated in the pathogenesis of metabolic syndrome, obesity and renal disease. Previous studies have revealed that high fat diets promote vascular calcification in uremic rats. Moreover, vitamin E has been shown to prevent uremic calcifications in genetically obese Zucker rats fed standard diet. The objective of this study was to investigate the influence of vitamin E supplementation on the development of extraskeletal calcifications in non-obese (wild type) uremic rats fed high fat diets.

**Methods:**

Wistar rats (*n* = 32) were preconditioned by feeding either a normal (NF) or high fat (HF) diet for 45 days and subsequently were subjected to 5/6 nephrectomy (Nx). Just before performing the first Nx step, a blood sample (Pre-Nx) was obtained. After Nx rats were switched to a diet with 0.9% phosphorus and supplemented with calcitriol. Also, after Nx, half of the rats from each group (NF and HF) were treated with vitamin E (VitE) in the diet (30,000 mg/kg) and the other half were maintained on basic VitE requirements (27 mg/kg). Thus, rats were allotted to four experimental groups: Nx-NF (*n* = 8), Nx-NF-VitE (*n* = 8), Nx-HF (*n* = 8) and Nx-HF-VitE (*n* = 8). At the time of sacrifice (day 66), blood and tissue samples were obtained.

**Results:**

Feeding a HF diet for 45 days did not increase body weight but elicited hyperglycemia, hypertriglyceridemia, an increase in plasma fibroblast growth factor 23 and a reduction in plasma calcitriol concentrations. After Nx, rats fed HF diet showed substantial extraskeletal calcification with aortic calcium content that was higher than in rats fed NF diet. Supplementation with VitE significantly (*p* < 0.05) reduced aortic (from 38.4 ± 8.8 to 16.5 ± 1.4 mg/g), gastric (from 5.6 ± 2.7 to 1.2 ± 0.4 mg/g) and pulmonary (from 1.8 ± 0.3 to 0.3 ± 0.2 mg/g) calcium content in rats on HF diets.

**Conclusions:**

Uremic rats fed HF diets developed more severe extraosseous calcifications than their normocaloric-fed counterparts and dietary VitE supplementation protected against uremic calcifications in rats fed HF diets. Thus, eating energy-rich foods should be discouraged in patients with renal disease and their deleterious effect may be ameliorated with adequate antioxidant supply.

## Background

Energy-dense food with a high fat content is implicated in the pathogenesis of obesity and metabolic syndrome (OB/MS), a significant and growing health problem both in Western and developing countries [1, 2]. The mechanisms involved in the pathogenesis of OB/MS are complex and include genetic predisposition, sedentary lifestyle and excesive intake of sugar- and fat rich foods [3, 4]. The relationship between OB/MS and renal disease is well known and can be explained by a variety of indirect mechanisms, including hypertension and type II diabetes [5, 6], and direct mechanisms, like increased glomerular capillary wall tension, changes in podocytes, down-regulation of the Sirt 1-adiponectin axis, etc. [7].

However, the influence of OB/MS on vascular calcifications (VC), one of the major contributors to cardiovascular mortality in patients with chronic kidney disease (CKD) [8], has not been studied with detail. When subjected to nephrectomy, obese Zucker rats have been reported to develop more severe extraskeletal calcifications than lean Zucker rats. In this experimental model, the influence of OB/MS on calcifications seems to be partly mediated by oxidative stress and calcifications can be prevented by treatment with vitamin E (VitE) [9].

Moreover, we have recently shown that uremic rats fed high fat (HF) diets also develop more severe VC than their counterparts fed normal fat (NF) diets. Phosphorus (P) retention, secondary to renal klotho and fibroblast growth factor 23 (FGF23) dysregulation, seems to be implicated in the procalcifying effect of HF diet [10]. In patients with CKD, elevated serum P plays a major role in the development of VC [11]. High extracellular P concentrations promote VC through a series of mechanisms, including phenotypic transdifferentiation of vascular smooth muscle cells (VSMCs) to osteogenic cells and increased serum calcium (Ca)xP product, which facilitates the deposition of Ca salts [12, 13]. Since high P also has been reported to promote oxidative stress in VSMCs [14], it would be important to determine whether antioxidant therapy with VitE is able to ameliorate calcifications in uremic rats fed a diet rich in fat.

We hypothesized that, as already shown in the model of genetic-obese Zucker rat, supplemental VitE might have a beneficial effect on uremic VC in non-obese wild type rats fed energy-dense diets. Thus, the objective of this study was to investigate the influence of VitE supplementation on uremic VC in rats fed HF diets.

## Methods

### Animals and surgical procedures

Three months-old Wistar rats (*n* = 32), provided by the Animal Housing Facilities of the University of Cordoba (Cordoba, Spain), were housed with a 12 h/12 h light/dark cycle, and given ad libitum access to a standard diet with normal Ca = 0.6% and P = 0.6%. Uremia was induced by 5/6 nephrectomy (Nx), a two-step procedure that reduces the original renal mass by five-sixths. Briefly, animals were anesthetized using xylazine (5 mg/kg, ip) and ketamine (80 mg/kg, ip). For the first step of the 5/6 Nx, a 5- to 8-mm incision was made on the left mediolateral surface of the abdomen. The left kidney was exposed, and the two poles (2/3 of renal mass) were ablated. The kidney was inspected and returned to an anatomically neutral position within the peritoneal cavity. The abdominal wall and skin incisions were closed with sutures, and the rat was placed back into its home cage. After 1 week of recovery, in the second step, the animal was reanesthetized and a 5- to 8-mm incision was made on the right mediolateral surface of the abdomen. The right kidney was exposed and unencapsulated, the renal pedicle was clamped and ligated, and the kidney was removed. The ligated pedicle was returned to a neutral anatomical position and the abdomen and skin incisions closed with suture materials. Fentanyl (0.2 mg/kg, ip) was used as analgesic agent. After the second surgery, the mineral content of the diet was changed to a diet containing Ca = 0.6% and P = 0.9% (Altromin Spezialfutter GmbH, Germany). Rats were supplemented with calcitriol, 80 ng/kg ip every other day (Calcijex, Abbot, Madrid, Spain). This dose of calcitriol has been previously shown to be effective in controlling secondary hyperparathyroidism. Independent of their P content, diets had either a normal fat content (NF diet) with a 5% fat concentration that provided Metabolizable Energy = 3518 kcal/kg (Altromin C 1000, Altromin Spezialfutter GmbH, Germany) or a high fat content (HF diet) with a 35% fat concentration that provided Metabolizable Energy = 5241 kcal/kg (Altromin C 1090–60, Altromin Spezialfutter GmbH, Germany). Sacrifice was performed by aortic puncture and exsanguination of the anesthetized (sodium thiopental, ip) rat. All experimental protocols were reviewed and approved by the Ethics Committee for Animal Research of the University of Cordoba (Cordoba, Spain). All protocols were carried out in accordance with the approved guidelines. They followed the guidelines laid down by the Higher Council of Scientific Research of Spain following the normal procedures directing animal welfare (Real Decreto 223/88, BOE of 18 of March) and adhered to the recommendations included in the Guide for Care and Use of Laboratory Animals (US Department of Health and Human Services, NIH) and European laws and regulations on protection of animals, under the advice of specialized personnel.

### Experimental design

**Fig. 1:**
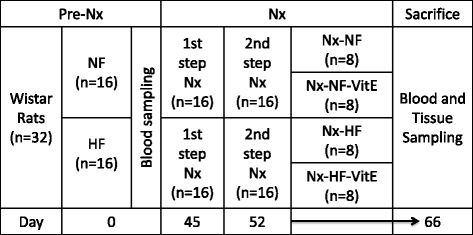
Diagram of the experimental design. Nx = 5/6 nephrectomy, NF = normal fat diet (5% fat), HF = high fat diet (35% fat), VitE = vitamin E added to the diet (30,000 mg/kg)

Sixteen rats were maintained for 45 days on NF diet and 16 rats on HF diet (both diets with 0.6% Ca and 0.6% P). At day 45, rats were subjected to Nx. Just before performing the first Nx step, a blood sample (Pre-Nx) was obtained from these 32 rats to assess the influence of the caloric content of the diet on metabolic status without the confounding effects of uremia. After the second step of Nx, at day 52, rats were switched to a 0.9% P diet and treated with calcitriol, as described above. The caloric content of the diets was maintained as before and the rats continued eating either NF or HF till the end of the experiments. Also, after Nx, half of the rats from each group (NF and HF) were supplemented with VitE in the diet (30,000 mg/kg) and the other half were maintained on basic VitE requirements (27 mg/kg). Thus, the four experimental groups were: Nx-NF (*n* = 8), Nx-NF-VitE (*n* = 8), Nx-HF (*n* = 8) and Nx-HF-VitE (*n* = 8). Rats were scheduled for sacrifice at day 80, four weeks after Nx, but due to the rapid deterioration experienced by the rats fed HF, sacrifice was performed at day 66 (Fig. 1).

### Assessment of vascular calcification

Following sacrifice, the thoracic aorta, stomach and lungs were dissected and processed to study mineral content. Vascular and soft tissue calcification was studied by histology and by measuring the tissue Ca content. Samples of the abdominal aorta, the right lung, and the stomach were fixed in 10% buffered formalin and subsequently sectioned and stained for mineralization by the Von Kossa method. Another portion of the aorta was demineralized in 10% formic acid, and the arterial tissue Ca content was measured in the supernatant. Quantification of tissue mineral content was performed as described previously [15]. Briefly, the stomach and the left lung from each rat were placed into separate 50-ml tubes. Twenty milliliters of 150 mM HCl was added to each tube. The tubes were mixed by inversion for 24 h at room temperature, and Ca was measured in the acid extract. Ca concentration in the acid extracts was measured by spectrophotometry (BioSystems SA, Barcelona, Spain).

### Blood chemistries

Blood for chemistry analyses was obtained either in anesthetized (inhaled sevofluorane) rats, from the jugular vein, or at the time of sacrifice (thiopental anesthesia plus exanguination), from the abdominal aorta. Blood for measurements of ionized Ca levels was collected in heparinized syringes and immediately analyzed using a Ciba-Corning 634 ISE Ca^2+^/pH Analyzer (Ciba-Corning, Essex, England). Afterwards, plasma was separated by centrifugation and stored at −20° C until assayed. Plasma creatinine, P, glucose, total cholesterol, high density lipoprotein (HDL) cholesterol, low density lipoprotein (LDL) cholesterol and triglycerides were measured by spectrophotometry (BioSystems SA, Barcelona, Spain). ELISA tests were used to quantify plasma FGF23 (Kainos Laboratories, Tokyo, Japan) and parathyroid hormone (PTH) (Immutopics, San Clemente, CA). Radioimmunoassay was used in plasma samples to determine insulin (Millipore, St. Charles, MO, USA), leptin (Millipore, St. Charles, MO, USA) and calcitriol (Immunodiagnostic Systems Ltd., Boldon, UK).

### Statistics

Values are expressed as the mean ± standard error. The difference between means for two different groups was determined by t-test; the difference between means for three or more groups was assessed by Analysis of Variance. Fisher Least Significant Difference test was used as a post-hoc procedure. A correlation study was carried out using the Pearson test. *p* < 0.05 was considered significant.

## Results

Before inducing uremia, body weight was similar in rats fed NF (198.8 ± 5.6 g) and in rats fed HF (202.5 ± 5.1 g). The rats that were fed HF diets did not increase body weight because they ingested less food: 10.7 ± 0.4 g/day than the rats fed NF diets: 13.0 ± 0.5 g/day. The differences in food intake between NF and HF diets were significant (*p* < 0.05). After Nx, body weight decreased both in rats fed NF (189.4 ± 3.5 g) and in rats fed HF (178.5 ± 3.1 g).

No differences in plasma creatinine were found between the rats that were fed NF and HF diets prior to Nx. However, rats fed HF diets had significantly higher glucose (134.6 ± 2.6 vs 125.4 ± 1.8 mg/dl), LDL cholesterol (6.6 ± 0.3 vs 5.0 ± 0.4 mg/dl) and triglycerides (72.9 ± 5.9 vs 50.3 ± 3.2 mg/dl). Plasma concentrations of FGF23 were higher in rats fed HF diet, 926 ± 121 pg/ml, than in rats fed NF diet, 217 ± 17 pg/ml, *p* < 0.05. No differences in plasma leptin and PTH were found between rats fed NF and HF diet. Prior to Nx, plasma concentrations of calcitriol were decreased in rats fed HF, 11.3 ± 1.2 pg/ml, when compared with rats fed NF, 77.7 ± 9.2 pg/ml, *p* < 0.001.

Nephrectomy resulted in an increase in creatinine in all experimental groups. At the time of sacrifice plasma creatinine was significantly higher in rats fed HF diet (1.8 ± 0.2 mg/dl) than in rats fed NF diet (1.3 ± 0.1 mg/dl). Treatment with VitE reduced plasma creatinine and the difference was significant in the rats fed NF diet (1.0 ± 0.1 mg/dl). Although plasma Ca was reduced in all uremic rats, higher values were recorded in the rats fed NF diet (1.14 ± 0.02 mmol/l). Plasma P was higher in the rats fed HF diet, 10.8 ± 1.4 mg/dl vs 6.6 ± 1.0 in the rats fed NF diet. VitE supplementation reduced P levels to 8.5 ± 0.5 mg/dl (HF diet) and 4.2 ± 0.4 mg/dl (NF diet). When compared with the non-uremic controls, Nx rats had higher glucose, insulin and cholesterol (total, LDL and HDL) concentrations. Plasma triglycerides were decreased in Nx groups and rats receiving supplemental VitE had the lowest triglycerides levels (*p* < 0.05 vs non-supplemented rats). As expected, the phosphaturic factor FGF23 was increased in all Nx groups and was higher in rats fed HF diets than in rats fed NF diets. Plasma PTH values, which in Nx rats were controlled by treatment with calcitriol, were not influenced by either diet caloric content or VitE supplementation. Uremic rats, that were treated with calcitriol, had mean plasma calcitriol concentrations ranging from 48.5 to 64.6 pg/ml and no significant differences were found between the four experimental groups (Table 1).

**Table 1:** Blood biochemistry from the study rats

	PreNx-NF	PreNx-HF	Nx-NF	Nx-NF-VitE	Nx-HF	Nx-HF-VitE
Creatinine (mg/dl)	0.6 ± 0.1^a^	0.5 ± 0.1^a^	1.3 ± 0.1^b^	1.0 ± 0.1^d^	1.8 ± 0.2^c^	1.5 ± 0.2^b^
Calcium (mmol/l)	1.30 ± 0.02^a^	1.29 ± 0.02^a^	1.14 ± 0.02^b^	1.04 ± 0.02^c^	1.01 ± 0.02^c^	1.00 ± 0.02^c^
Phosphorus (mg/dl)	3.9 ± 0.4^a^	4.1 ± 0.2^a^	6.6 ± 1.0^a^	4.2 ± 0.4^a^	10.8 ± 1.4^b^	8.5 ± 0.5^b^
Glucose (mg/dl)	125.4 ± 1.8^a^	134.6 ± 2.6^b^	167.8 ± 5.6^c^	165.3 ± 5.9^c^	158.2 ± 6.4^c^	145.0 ± 2.3^b^
Insulin (ng/ml)	0.21 ± 0.04^a^	0.16 ± 0.02^a^	0.75 ± 0.18^b^	0.52 ± 0.09^b^	0.32 ± 0.14^a^	0.71 ± 0.21^b^
Total Cholesterol (mg/dl)	60.2 ± 2.2^a^	64.5 ± 3.0^a^	114.4 ± 6.6^b^	68.8 ± 6.8^a^	113.2 ± 5.4^b^	117.5 ± 10.6^b^
LDL Cholesterol (mg/dl)	5.0 ± 0.4^a^	6.6 ± 0.3^b^	7.6 ± 1.3^b^	4.6 ± 0.6^a^	9.6 ± 1.6^c^	10.5 ± 2.1^c^
HDL Cholesterol (mg/dl)	23.1 ± 1.0^a^	25.9 ± 1.9^a^	44.6 ± 2.0^b^	32.8 ± 2.2^c^	48.8 ± 3.7^b^	44.5 ± 4.4^b^
Triglycerides (mg/dl)	50.3 ± 3.2^a^	72.9 ± 5.9^b^	32.6 ± 3.2^c^	14.1 ± 1.2^d^	35.2 ± 3.5^c^	48.3 ± 10.7^a^
FGF23 (pg/ml)	217 ± 17^a^	926 ± 121^b^	8984 ± 2333^c^	5055 ± 1588^c^	20492 ± 3906^d^	24579 ± 7879^d^
Leptin (ng/ml)	1.4 ± 0.2^a^	1.4 ± 0.2^a^	0.7 ± 0.3^b^	1.4 ± 0.4^a^	1.8 ± 0.4^a^	0.8 ± 0.5^a^
PTH (pg/ml)	70.9 ± 7.6^a^	55.1 ± 2.5^a^	59.4 ± 3.9^a^	59.3 ± 4.2^a^	64.3 ± 6.9^a^	71.1 ± 14.2^a^
Calcitriol (pg/ml)	77.7 ± 9.2^a^	11.3 ± 1.2^b^	54.3 ± 8.7^a^	48.5 ± 6.9^a^	64.6 ± 5.1^a^	48.7 ± 12.9^a^

For each parameter, values with different superscript are significantly (*p* < 0.05) different

**Fig. 2:**
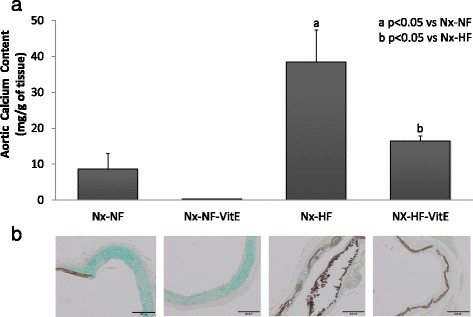
**a** Calcium content (mg/g of tissue) in the aortas of rats from the four experimental groups. **b** Representative von Kossa stained aortic tissue sections from the same rats where mineral deposits are depicted by the brown pigment. Nx-NF = uremic rats treated with calcitriol (80 ng/kg ip eod) fed normal fat diet with 0.9% phosphorus (*n* = 8), Nx-NF-VitE = uremic rats treated with calcitriol (80 ng/kg ip eod) fed normal fat diet with 0.9% phosphorus and supplemented with vitamin E, 30,000 mg/kg (*n* = 8), Nx-HF = uremic rats treated with calcitriol (80 ng/kg ip eod) fed a high fat diet with 0.9% phosphorus (*n* = 8), Nx-HF-VitE = uremic rats treated with calcitriol (80 ng/kg ip eod) fed high fat diet with 0.9% phosphorus and supplemented with vitamin E, 30,000 mg/kg (*n* = 8). a *p* < 0.05 vs Nx-NF, b *p* < 0.05 vs Nx-HF

Rats fed HF diet showed substantial aortic calcification with calcium content that was significantly higher than in rats fed NF diet (38.4 ± 8.8 mg/g tissue vs 8.7 ± 4.2 mg/g tissue, *p* < 0.001). Supplementation with VitE reduced aortic calcium content in rats on HF diets to 16.5 ± 1.4 mg/g tissue (*p* = 0.018). Differences in aortic calcification between experimental groups were also evident by histology with Von Kossa staining (Fig. 2).

**Fig. 3:**
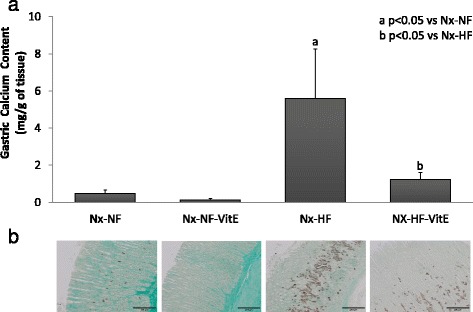
**a** Calcium content (mg/g of tissue) in the stomachs of rats from the four experimental groups. **b** Representative von Kossa stained gastric tissue sections from the same rats where mineral deposits are depicted by the brown pigment. Nx-NF = uremic rats treated with calcitriol (80 ng/kg ip eod) fed normal fat diet with 0.9% phosphorus (*n* = 8), Nx-NF-VitE = uremic rats treated with calcitriol (80 ng/kg ip eod) fed normal fat diet with 0.9% phosphorus and supplemented with vitamin E, 30,000 mg/kg (*n* = 8), Nx-HF = uremic rats treated with calcitriol (80 ng/kg ip eod) fed a high fat diet with 0.9% phosphorus (*n* = 8), Nx-HF-VitE = uremic rats treated with calcitriol (80 ng/kg ip eod) fed high fat diet with 0.9% phosphorus and supplemented with vitamin E, 30,000 mg/kg (*n* = 8). a *p* < 0.05 vs Nx-NF, b *p* < 0.05 vs Nx-HF

**Fig. 4:**
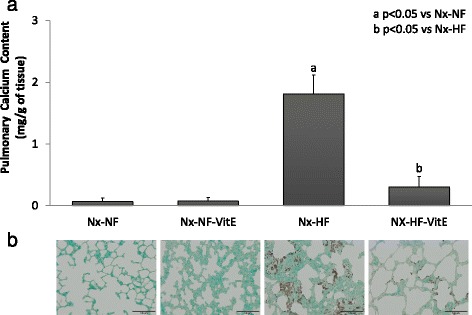
**a** Calcium content (mg/g of tissue) in the lungs of rats from the four experimental groups. **b** Representative von Kossa stained pulmonary tissue sections from the same rats where mineral deposits are depicted by the brown pigment. Nx-NF = uremic rats treated with calcitriol (80 ng/kg ip eod) fed normal fat diet with 0.9% phosphorus (*n* = 8), Nx-NF-VitE = uremic rats treated with calcitriol (80 ng/kg ip eod) fed normal fat diet with 0.9% phosphorus and supplemented with vitamin E, 30,000 mg/kg (*n* = 8), Nx-HF = uremic rats treated with calcitriol (80 ng/kg ip eod) fed a high fat diet with 0.9% phosphorus (*n* = 8), Nx-HF-VitE = uremic rats treated with calcitriol (80 ng/kg ip eod) fed high fat diet with 0.9% phosphorus and supplemented with vitamin E, 30,000 mg/kg (*n* = 8). a *p* < 0.05 vs Nx-NF, b *p* < 0.05 vs Nx-HF

Mineral deposition in stomach and lung was less severe than in aorta and only was obvious in rats fed HF. Thus Nx-HF rats had a calcium content of 5.6 ± 2.7 mg/g in the stomach and 1.8 ± 0.3 mg/g in the lungs. Dietary VitE supplementation resulted in a significant decrease in soft tissue calcification both in stomach, 1.2 ± 0.4 mg/g (*p* = 0.024), and in the lungs, 0.3 ± 0.2 mg/g (*p* < 0.001). Von Kossa staining of stomach and lung tissue sections also demonstrated the presence of calcifications in rats fed HF and their attenuation with dietary vitamin E (Figs. 3 and 4).

## Discussion

This study was designed to investigate the influence of supplementing VitE on the development of extraskeletal calcifications in uremic rats fed diets with HF content. Our results confirm a previous report of a pro-calcifying effect of HF diets in uremic rats and demonstrate that dietary VitE supplementation protects against uremic calcifications in rats fed HF diets.

During the study the rats fed HF adapted food consumption to maintain a caloric intake similar to rats fed NF (around 50 kcal/day), thus no increase in body weight and plasma leptin concentrations was observed in rats fed HF. This allowed to study the effect of the fat content of the diet independent of obesity-mediated mechanisms (e.g. adipokines and other inflammatory mediators released by fat tissue). At the time of Nx the animals fed HF diet were not obese but they showed biochemical signs of metabolic syndrome: hyperglycemia, hypertriglyceridemia and increased LDL. At this stage, with a background of metabolic changes induced by high caloric intake, we had the opportunity to explore the impact of renal failure on VC and the influence of VitE.

The dose of vitamin E used in the experiments was chosen based on previous studies [9]. Rats ingested around 300 mg of vitamin E per day which is the upper limit of what is considered safe for long term administration of vitamin E to humans [16]. Although it is difficult to extrapolate the effect of this dose of vitamin E to humans, none of the side effects described in people after vitamin E overdose (diarrhea, skin inflammation, hyperglycemia, hyperlipidemia) were observed in the present study.

Our results confirm previous data showing that preconditioning with HF diet predisposes rats to develop extraskeletal calcifications associated to renal failure [10]. This finding highlights the potential deleterious effect on VC of energy-dense diets, even in the absence of obesity. Moreover, treatment with VitE reduced the severity of extraskeletal calcifications in rats fed HF. VitE has been previously shown to be effective in preventing VC in rats with genetic obesity [9]. The results of the present study expand these data to a population of non-obese, although metabolically challenged, rats and demonstrate that obesity per se is not a requisite for the deleterious effect of HF diets on VC in uremic rats. When compared with a earlier report in which genetic obese Zucker rats fed a NF diet were subjected to the same experimental protocol, VC was much more severe in the wild type rats fed HF diet of the current study [9]. In fact, the present study was originally designed to last 4 weeks after Nx (same time frame than the previous study with Zucker rats) but the rapid deterioration of the rats fed HF diets required to terminate the study, for humane reasons, 14 days after Nx.

Although it was not a primary objective of this study, the data obtained also provide interesting insights about the role of calcitriol on VC in the context of feeding HF diets. Prior to Nx, rats fed HF diets had very low plasma calcitriol concentrations. A decrease in plasma calcitriol, without changes in plasma calcidiol, has been previously reported in rats with normal renal function after feeding HF diets [10]. Since FGF23 is known to decrease calcitriol synthesis through inhibition of 1-alpha-hydroxylase activity in the kidney [17], these low calcitriol concentrations have been linked to the increase in FGF23 elicited by HF diets [10]. In uremic rats calcitriol should be further reduced by the decrease in renal mass and by the elevation in FGF23 secondary to kidney disease [18]. Vitamin D deficiency is associated with cardiovascular disease [19–21] and calcitriol treatment has been reported to improve survival of CKD patients [22, 23]. Thus, it could be speculated that treatment with calcitriol may have beneficial effects on uremic rats fed HF. However, when comparing the results of this study with previous data in which rats fed HF were not treated with calcitriol [10], calcifications were much more severe after calcitriol treatment. Therefore, the results of this study demonstrate that even though rats fed HF diets have very low plasma calcitriol concentrations, treatment with calcitriol at doses sufficient to control secondary hyperparathyroidism is clearly deleterious. The negative actions of calcitriol are probably related to an increased P load in animals with impaired P excretion derived from feeding HF diets [10], which would override any beneficial effect that restoring plasma calcitriol concentrations may have on vascular health. In fact, the data of this study raise the intriguing question of whether the decrease in plasma calcitriol observed after feeding HF diets might act as a protective mechanism in rats that already have difficulty in excreting P.

The decrease in phosphate levels in uremic rats treated with vitamin E is probably related to the reduction in extraskeletal calcification: nephrocalcinosis was less severe in animals treated with vitamin E thus allowing their remnant kidneys to be more efficient in phosphate excretion.

The mechanisms to explain the detrimental effect of HF diets on VC are probably multifactorial. In previous studies with obese Zucker rats we have shown that oxidative stress plays an important role in obesity-associated VC [9]. The results of the present study also support a role of oxidative stress in HF diet-induced calcifications, because treatment with VitE substantially decreased VC. In addition to any direct pro-oxidant action of feeding HF, HF diet-induced P retention may also contribute to oxidative stress [14]. Thus, VitE treatment may help to modulate the adverse pro-oxidant effects of P retention secondary to feeding HF. Nonetheless since the study was performed in an animal model of uremia, caution should be taken when extrapolating the results to the human situation.

## Conclusions

In conclusion, uremic rats fed HF diets develop more severe extraosseous calcifications than their normocaloric-fed counterparts and dietary VitE supplementation protects against uremic calcifications in rats fed HF diets. Thus, eating energy-rich foods should be discouraged in patients with renal disease and their deleterious effect may be ameliorated with adequate antioxidant supply.

## Abbreviations

Ca: Calcium
CKD: Chronic Kidney Disease
FGF23: Fibroblast Growth Factor 23
HDL: High Density Lipoprotein
HF: High Fat
LDL: Low Density Lipoprotein
NF: Normal Fat
Nx: Nephrectomy
OB/MS: Obesity/Metabolic Syndrome
P: Phosphorus
PTH: Parathyroid Hormone
VC: Vascular Calcification
VitE: Vitamin E
VSMCs: Vascular Smooth Muscle Cells
